# Amiodarone prevents wave front-tail interactions in patients with heart failure: an in silico study

**DOI:** 10.1152/ajpheart.00227.2023

**Published:** 2023-09-01

**Authors:** Richard A. Gray, Michael R. Franz

**Affiliations:** ^1^Division of Biomedical Physics, Office of Science and Engineering Laboratories, Center for Devices and Radiological Health, Food and Drug Administration, Silver Spring, Maryland, United States; ^2^Cardiology Division, Veteran Affairs Medical Center, Washington, District of Columbia, United States; ^3^Department of Pharmacology, Georgetown University Medical Center, Washington, District of Columbia, United States

**Keywords:** action potential, amiodarone, antiarrhythmic drugs, computer simulation, heart failure

## Abstract

Amiodarone (AM) is an antiarrhythmic drug whose chronic use has proved effective in preventing ventricular arrhythmias in a variety of patient populations, including those with heart failure (HF). AM has both class III [i.e., it prolongs the action potential duration (APD) via blocking potassium channels) and class I (i.e., it affects the rapid sodium channel) properties; however, the specific mechanism(s) by which it prevents reentry formation in patients with HF remains unknown. We tested the hypothesis that AM prevents reentry induction in HF during programmed electrical stimulation (PES) via its ability to induce postrepolarization refractoriness (PRR) via its class I effects on sodium channels. Here we extend our previous human action potential model to represent the effects of both HF and AM separately by calibrating to human tissue and clinical PES data, respectively. We then combine these models (HF + AM) to test our hypothesis. Results from simulations in cells and cables suggest that AM acts to increase PRR and decrease the elevation of takeoff potential. The ability of AM to prevent reentry was studied in silico in two-dimensional sheets in which a variety of APD gradients (ΔAPD) were imposed. Reentrant activity was induced in all HF simulations but was prevented in 23 of 24 HF + AM models. Eliminating the AM-induced slowing of the recovery of inactivation of the sodium channel restored the ability to induce reentry. In conclusion, in silico testing suggests that chronic AM treatment prevents reentry induction in patients with HF during PES via its class I effect to induce PRR.

**NEW & NOTEWORTHY** This work presents a new model of the action potential of the human, which reproduces the complex dynamics during premature stimulation in heart failure patients with and without amiodarone. A specific mechanism of the ability of amiodarone to prevent reentrant arrhythmias is presented.

## INTRODUCTION

Sudden cardiac death due to ventricular arrhythmias is a major cause of mortality in patients with heart failure (HF). It has been shown that HF increases the vulnerability to reentrant tachyarrhythmias (1–3). A common trigger for these reentrant arrhythmias is premature ventricular beats, which was the reason for the once popular use of programmed electrical stimulation (PES) in the clinical laboratory (4). Why are premature depolarizations so effective in causing these arrhythmias? We previously have shown in vivo, using monophasic action potential (MAP) recordings in patients, that tightly coupled, repetitive premature action potentials, whose wave “front” encroached onto the preceding final repolarization (wave “tail”), exhibit a significantly lower upstroke velocity, as well as, decreased conduction velocity (CV) as compared with action potentials (APs) elicited later during the diastolic interval, when they arise from fully restored resting potential (5, 6). The encroachment of a premature AP wavefront onto the relatively refractory tail of the preceding wave, which equals a decrease in takeoff potential (TOP), is of paramount importance to reentry induction, which also has been documented in the human in vivo heart (6–8). The interval from the end of absolute refractoriness, when the first slow-upstroke AP can be induced, to the end of relative refractoriness, when the latest stimulus-induced AP before full recovery can occur, is considered the “vulnerable period” or “vulnerable window.”

In striking contrast, when amiodarone (AM) was administered to inducible patients during PES, it caused an increase in action potential duration (APD) prolongation and, in addition, postrepolarization refractoriness (PRR) (6); PRR is the difference between the effective refractory period (ERP) and APD. PRR prevented early, “encroaching” depolarizations, and thus the earliest possible depolarizations occurred outside the relative refractory period and exhibited normal upstrokes and CV. We suggest that AM’s unique effect on the rapid sodium current results in significant PRR and thereby decreases wave front-tail interactions and prevents the initiation of reentry. This is supported by the clinical findings of Osaka et al. (9) who demonstrated that AM suppressed VT/VF in patients with structural heart disease and this was concomitant with an increase in the shortest diastolic interval to produce a ventricular response. Our suggestion is also informed by our previous studies (6, 7); in fact, Franz et al. (6) have stated that AM “appears to have unique characteristics in that it produces PRR, followed by rapid voltage-dependent recovery of the fast sodium channel. Once PRR gives way to reexcitability, more normal premature responses than in the drug-free state may be elicited.”

**Figure 1.**
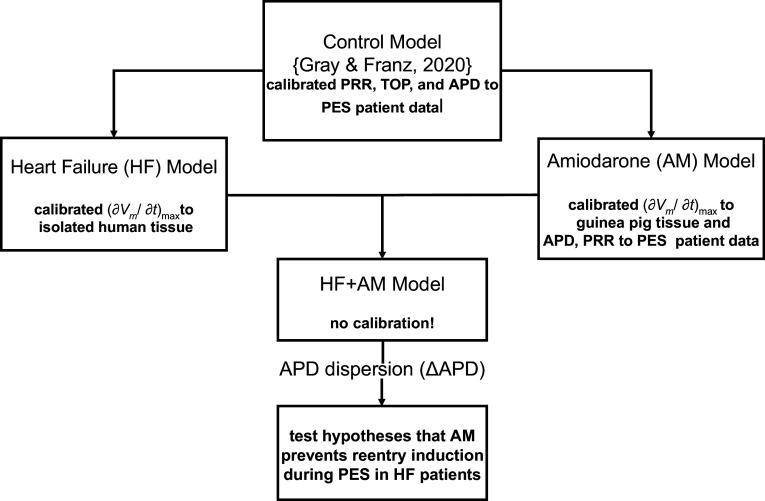
Overview of computational model approach including development and simulations. Original (control) model was modified to represent the effects of heart failure (HF) and amiodarone (AM) separately, and these models were combined to represent patients with HF during chronic AM treatment (HF + AM). The HF + AM model was studied in two-dimensional sheets to test the hypothesis that AM prevents reentry induction during programmed electrical stimulation (PES) in patients with HF primarily by inducing postrepolarization refractoriness (PRR) via alteration of rapid sodium channel.

AM has proved effective in preventing ventricular arrhythmias in a wide range of patient groups including coronary heart disease, ischemic heart disease, and structural heart disease (6, 9–11), however, the specific mechanism(s) by which it prevents reentry formation in HF remains unknown. Here, we use computational modeling to test the hypothesis that AM prevents reentry induction during PES in patients with HF, primarily by inducing PRR via altering rapid sodium channel inactivation and preventing wave front-tail interactions. We extend our previous human action potential model calibrated to APs recorded from patients undergoing PES (12) to develop action potential models of HF and AM, which were individually calibrated and validated, and then combined (HF + AM model) to test our hypothesis (see Fig. 1).

Computational modeling is an excellent approach to test this hypothesis because it allows idealized control and implementation of the multifactorial phenomena of the complex physiology of wave front-tail interactions, drug effects, tissue heterogeneity, cardiac memory, refractory period and PRR, vulnerable period and reentry initiation, etc., as described below (see *Model Motivation*). Despite numerous studies demonstrating the clinical effectiveness of the antiarrhythmic properties of AM (6, 9–11), there is a dearth of corresponding AP and conduction measurements in such patients making model calibration difficult. Fortunately, clinical recordings from our previous study (6) were made using the Franz MAP-pacing combination catheter (13), which allows simultaneous measurement of both ERP and APD (and therefore PRR) at a single site. This allowed us to develop and calibrate the AM models using the values of PRR measured from patients during PES. To allow comparison with other antiarrhythmic drugs, we introduce a model of a “pure” class III drug d-sotalol also calibrated to the PRR data from Franz et al. (6). As we demonstrate below, computer simulation results suggest that AM acts on the AP to increase PRR and decrease the elevation of TOP during PES resulting in the prevention of reentry induction in two-dimensional (2-D) sheets of tissue exhibiting heterogeneous APD.

## METHODS

### Model Motivation

We tested the ability of AM to prevent reentry formation in HF by simulating 2-D sheets of isotropic tissue in response to S1–S4 stimuli. The HF, AM, and HF + AM action potential models were developed by extending our previous human action potential model (12); all equations here are identical to the original model, only parameter values were changed. The models are designed to be as simple as possible and consistent with clinical and voltage-clamp data while minimizing the number of free parameters determined by calibration as done previously (12). The model was developed to accomplishes a desired level of explanation or prediction (specifically, model calibration included APD and CV at baseline as well as PRR measured from patients during PES) with as few parameters as possible. HF was modeled using the well-known effects on ionic currents (14–17). To represent the complexity of AM pharmacology, including differential acute and chronic effects (18–21) and metabolism (22), and limited and disparate voltage-clamp data, we constructed eight AM model “variants” by combining effects on various parameters. In addition to characterizing the effects of AM, we also developed a model of a drug with class III properties, d-sotalol (DS), that could be calibrated similarly to AM and allow a comparison of drug effects. Importantly, the AM and DS models were calibrated using PRR values measured from patients during PES (6), which makes the results clinically relevant. All models are described below, and the parameters are provided in Supplemental Tables S1 and S2 (note: all Supplemental tables and figures may be found at https://doi.org/10.6084/m9.figshare.24065610). The inclusion of tissue heterogeneity is problematic because there are literally an infinite number of implementation possibilities. Although the specific spatial patterns of APD in patients with HF with and without AM are largely unknown and probably complex (3, 23, 24), we chose an idealized implementation of linear spatial gradients to represent a variety of effects such as heterogeneities of drug effects as well as ionic channel and disease expression. We introduced 2-D pyramidic gradients such that the boundary elements were represented using the control (HF) model and the center element was represented with the HF (HF + drug) model with a linear gradient of g_C,aL_ in the vertical direction and potassium conductances in the horizontal direction. This approach introduces a level of “robustness” because each 2-D simulation actually incorporates thousands of unique cell action potential models, for example, the medium tissue simulations are comprised of 1,000 × 1,000 cells corresponding to 62,500 unique model parameterizations due to the pyramidic symmetry. Since both the APD gradient and the size of tissue is important in regard to the ability to induce sustained reentry (25), we performed 2-D simulations in three square tissue sizes [length = 16, 20, and 24 cm with the largest, approximately representing the surface area of the human heart (26), assuming an anisotropic ratio of 2.5], each with a unique APD gradient.

### Heart Failure Model

HF remodeling of repolarization currents was implemented by decreasing peak potassium currents: *I*_Ks_ by 58% (27), *I*_K1_ by 41% (27), *I*_to_ by 44% (27), and *I*_Ca,L_ by 25% (28). We adjusted peak *I*_Na_ by decreasing g_Na_ by 39% to match the value of d*V*/d*t*_max_ of 180 mV/ms measured from isolated human tissue from patients with HF (29). The HF model parameter values are provided in Supplemental Table S1. These alterations resulted in an increase of APD from the control value of 288 to 320 ms at a BCL = 1,000 ms.

### Amiodarone Models

The effects of AM are difficult to characterize, in part because of the differences between chronic and acute effects. We chose to implement the effects of AM using eight AM model “variants” (AM1–AM8) to depict the disparate experimental data. First, we chose to implement AM effects on repolarization currents according to the ratio of peak current block measured from chronic exposure of AM on guinea pig myocytes (30) of *I*_Ks_ by −39%; *I*_K1_ −64%; and *I*_Kr_ −55%. Since some studies (31) have shown no effect on *I*_K1_, we also included models with no effect of AM on *I*_K1_. The absolute values of these three (or two) potassium conductances were uniformly adjusted to achieve an APD of 298 ms at BCL = 400 ms to match the 23% increase measured by Franz et al. (6). This conductance scaling factor was 1.4 for a 64% decrease in *I*_K1_ and was 1.45 when *I*_K1_ was not reduced. We could find no voltage-clamp studies on the chronic effect of AM on *I*_Na_, but did identify one chronic study that demonstrated a decrease of 16.4% in d*V*/d*t*_max_ (32). Therefore, we adjusted g_Na_ to achieve this decrease (from 245 mV/ms in control to 205 mV/ms) except for variants AM5 and AM6 in which we assumed that d*V*/d*t*_max_ was unaltered. We found two studies of the acute effect of AM illustrating a shift of the availability curve to more hyperpolarized values, –15 mV (33) and −5.9 mV (34). However, implementing a shift of −15 mV (only) prevented propagation, therefore we decreased this magnitude to −10.8 mV and did not concomitantly decrease g_Na_ because it resulted in conduction failure. We identified one study of the acute effect of AM showing a slowing of the recovery of *I*_Na_ inactivation (33). All together the combination of these changes resulted in eight variants which were calibrated by simultaneously adjusting g_Na_ and the time constant of *I*_Na_ inactivation (TauH) to achieve the desired values of (∂*V*/∂*t*)_max_ (either 245 mV/ms or 205 mV/ms) and PRR of ∼8 ms for S2, S3, and S4 beats measured by Franz et al. (6) in the RV of patients with AM. The AM model parameter values are provided in Supplemental Table S2. Since all the AM variants included a significant slowing of *I*_Na_ inactivation (12.78 to 22.72 times, see Supplemental Table S2), we also modified all eight AM variants by reducing the magnitude of this change study this effect (see below).

### d-Sotalol Model

We implemented the effects of d-sotalol (DS) to match the clinical results of Franz et al. (6). Specifically, we altered the control model (12) by simultaneously decreasing the conductance of *I*_Kr_ by 23% to achieve an APD of 265 ms at BCL = 400 ms to match the 11% increase in (6), and increasing the time constant of *I*_Na_ inactivation (TauH) by a factor of 2.0 to match the PRR values in patients with DS (6). The voltage dependence of TauH for all models is shown in Supplemental Fig. S1.

### Heart Failure with Amiodarone Models

The HF and AM as well as the HF and DS models were combined assuming their effects are independent such that the effects on conductances were the product of individual effects. This resulted in eight HF + AM model variants representing the effects of HF and both the class I and III effects of AM and one HF + DS model. A sensitivity analysis of how model parameters alterations from the original model affect PRR, ΔTOP for cell and (∂*V*/∂*t*)_max_ and CV for cable simulations is presented in the Supplemental Table S3.

### Virtual Protocols

Virtual S1–S4 stimulation was performed in single cells and cables as done previously (12) to mimic the clinical scenario of programmed electrical stimulation (PES) (5). Briefly, eight S1 pacing beats at 400 ms were applied (stimulus duration of 2 ms with amplitude of −30 pA/pF, which is approximately twice threshold), and the effective refractory period (ERP) was then determined for three premature beats (S2, S3, and S4) using 1-ms increments (5 ms in 2-D). Suprathreshold activation was defined using the following criteria: peak sodium current more negative than −100 µA/cm^2^ for single-cell simulations and propagation along the entire tissue for 1-D and 2-D simulations. Transmembrane potential thresholds for depolarization time and repolarization times were −30 and −75 mV, respectively. This induction protocol is similar to the virtual heart arrhythmia risk prediction (VARP) of Prakosa et al. (35) applied to virtual patient-specific hearts. Briefly, eight S1 pacing beats at 400 ms were applied from the bottom left corner of the sheet, then ERP was determined at the same site for three premature beats (S2, S3, and S4). Various 2-D APD gradients were introduced in simulations similar to the one in Ref. 36 as follows. The gradients were implemented via triangular gradients of both gCaL in the vertical direction and potassium currents in the horizontal direction to ensure horizontal and vertical asymmetry such that the largest APD occurred near the center of the sheet. The values of conductances at the bottom left and upper right corner were equal to control values and the values at the center were equal to either HF or HF + AM model values.

### Numerical Methods

Computer simulations were performed using custom software developed using PVwave (Visual Numerics). All equations were integrated using the forward Euler method with time step equal to 0.001 ms for single cells and 1-D cable (5 cm) simulations and 0.01 ms for 2-D simulations. Spatial discretization was 0.01 cm in 1-D and 0.02 in 2-D; all tissue simulations were isotropic with a diffusion coefficient of 0.001 cm/ms^2^ and CV was computed in quiescent tissue. As done previously (12), the initial conditions for all simulations were the same initial conditions (initial transmembrane membrane potential of −88 mV, gating variables equal to the corresponding steady-state values at −88 mV, and diastolic calcium equal to zero) to ensure reproducibility, the diastolic values of all variables equilibrated during steady-state pacing at BCL = 400 ms.

## RESULTS

### Baseline Behavior

**Figure 2.**
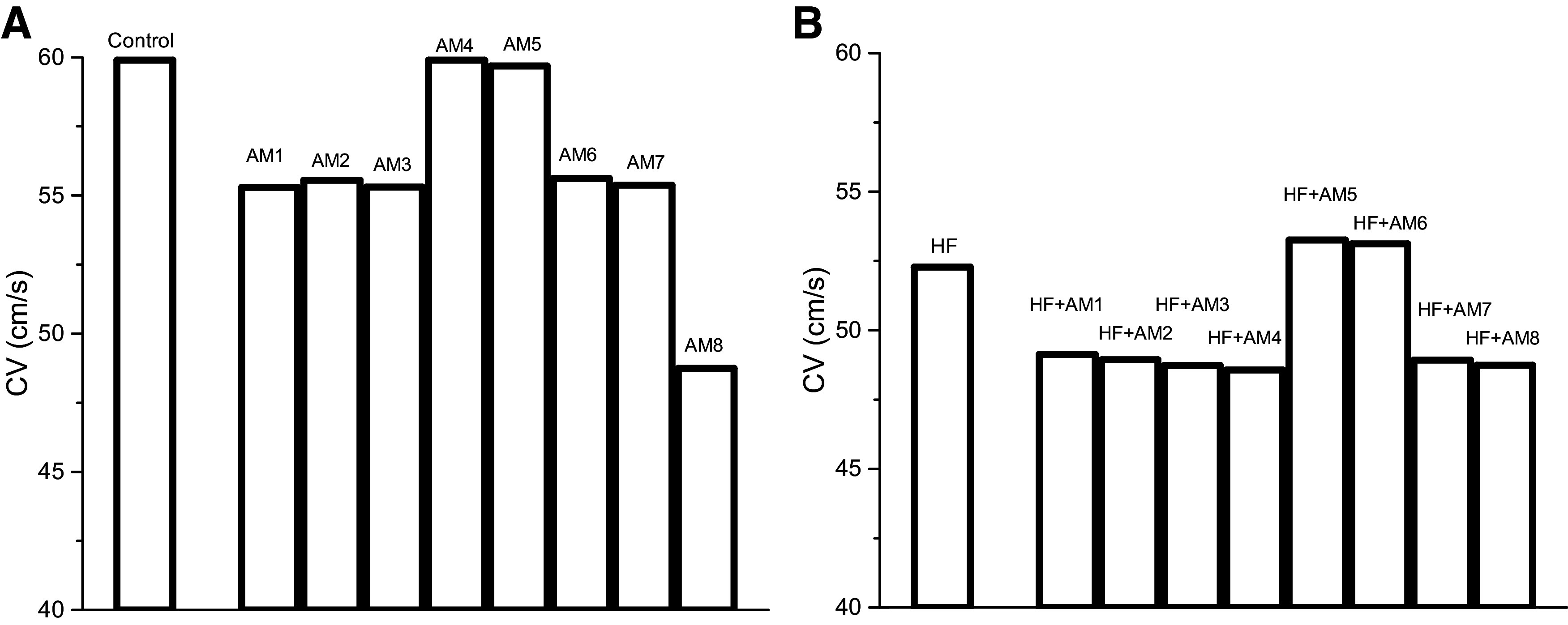
*A* and *B*: conduction velocity (CV) for control and 8 AM models (*A*) and heart failure (HF) and 8 HF + amiodarone (AM) models (*B*).

The APD at BCL = 400 ms in the cellular and cable simulations were very similar for all models (i.e., AM, HF, and HF + AM). APD in the cable for AM models were similar to the cellular APD value of 298 used for calibration and ranged from 298 to 301 ms; APD in the cable for HF was 276 ms and the cellular APD was 279 ms; and APD in the cable for HF + AM ranged from 363 to 367 and were similar to the cellular APD values of 361 to 364 ms. The maximum CV values for all the models are shown in Fig. 2. CV varied in the model variants with the highest values occurring for the models in which (∂*V*/∂*t*)_max_ was not altered from control (AM5 and AM6) or HF (HF + AM5 and HF + AM6) values.

### Model Validation

Here we perform model validation by comparing baseline simulation results to clinical and experimental data. The APD in our HF model (BCL = 1,000 ms) was increased by 11% compared with control, which is similar to that measured in isolated human tissue of 16–18% (29, 37). The decrease of peak *I*_Na_ by 39% for the HF model reduced CV by 13% compared with control, which is consistent with the results from the isolated rabbit heart (2, 38). The diastolic calcium level in our HF model was the same as control for BCL = 2,000 ms consistent with that measured in the rabbit (39), but was increased by a factor of 3 at 3 Hz similar to that observed in the rabbit (x2.3) in isolated rabbit cells (40). The APD in our AM models (BCL = 1,000 ms) was increased by 32% compared with control; the data from published studies vary over a wide range from ∼10 to 75% (7, 41, 42). The decrease in CV for our eight AM models compared with control ranged from 0 to 19% which spans the range reported in the literature (7, 9, 41, 43). The increase in *DI_min_* for virtual S1–S2 pacing in a cable at a BCL of 400 ms from control to AM ranged from 22.4 to 25.3 ms and from HF to HF + AM ranged from 18.3 to 21.8 ms consistent with clinical results (9). The increase in cellular APD was 44% for HF + AM compared with HF, which is similar to some clinical measurements of QT intervals (44) but larger than in others (9, 45, 46).

### Virtual Programmed Electrical Stimulation Results

**Figure 3.**
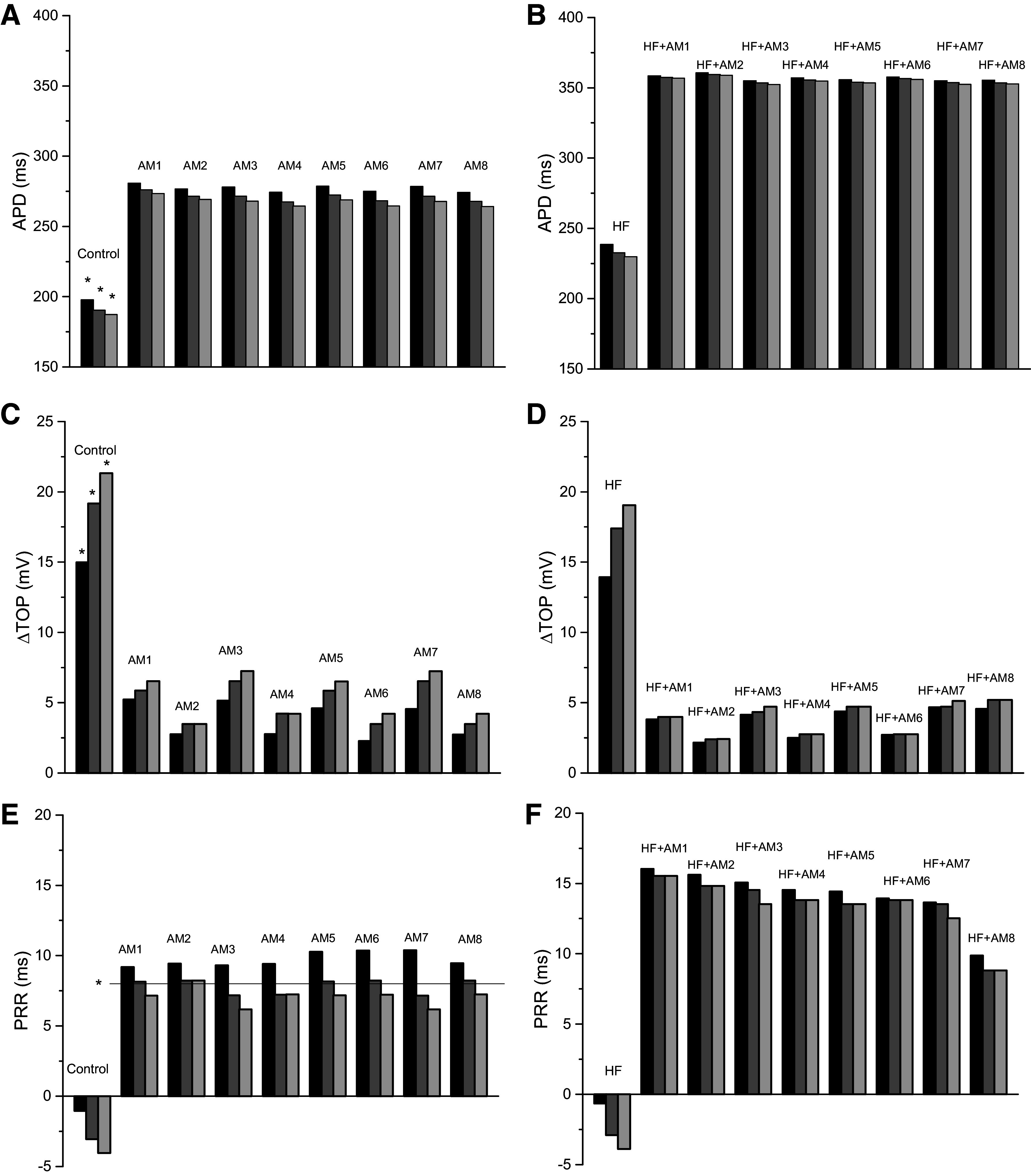
Results from cellular action potential simulations (S2, black; S3, dark gray; S4, light gray): control model and 8 amiodarone (AM) model variants (*left*); heart failure (HF) model and 8 HF + AM model variants (*right*). *A–F*: action potential duration (APD; *A* and *B*), change in take-off potential (ΔTOP; *C* and *D*), and post-repolarization refractoriness (PRR; *E* and *F*). Corresponding results from cable simulations are presented in Supplemental Fig. S2.

**Figure 4.**
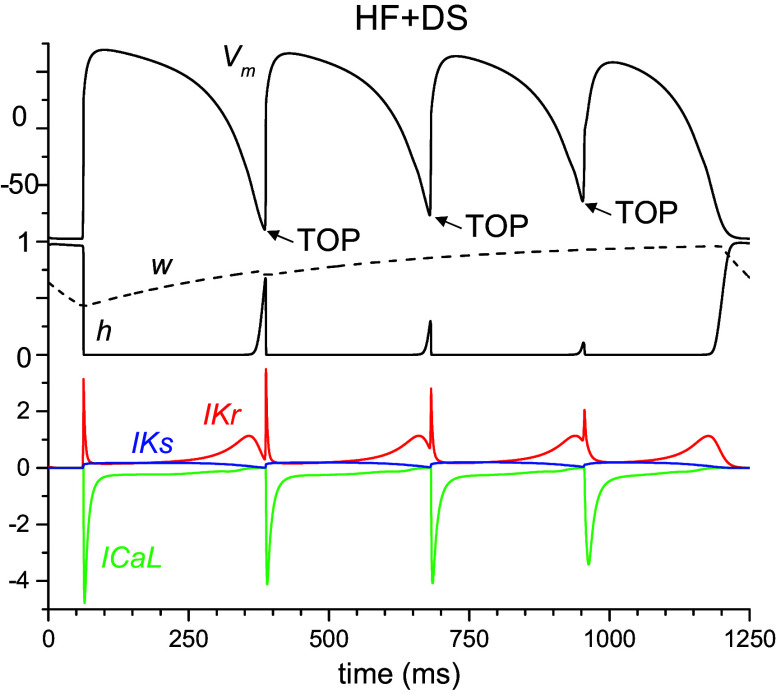
Results from cable simulations: transmembrane potential (*V*_m_; in mV), gating variables (*w* and *h*) dimensionless, and *I*_ion_ currents (*I*_Ca,L_, *I*_Ks_, *I*_Kr_; in μA/cm^2^) during S1–S4 for heart failure (HF) + d-sotalol (DS) model. The values of peak *I*_Na_ were S1: −322; S2, −264; S3, −150; and S4, −62 μA/cm^2^; this progressive change in *I*_Na_ is a result of the progressive decrease in the value of the *h* gate. Arrows indicate the decrease of the takeoff potential (TOP) during programmed electrical stimulation (PES).

The results from the virtual programmed electrical stimulation (PES) protocols in single cells for all models are shown in Fig. 3; the corresponding results from cables are presented in the Supplemental Fig. S2. APD decreased progressively during the S1–S4 beats for control and all AM models (Fig. 3*A*) as well as for HF and all HF + AM models (Fig. 3*B*). The values of ΔTOP for the AM models were less than control and the values of ΔTOP for the HF + AM models were less than for HF; however, the progression during S2–S4 was less (Fig. 3*D*). The progression of both APD and ΔTOP during S2–S4 in the cable simulations (Supplemental Fig. S2) was considerably greater than in individual cells indicating complex front-tail dynamics during propagation. PRR in AM simulations varied little for beats S2–S4 (Fig. 3*E*) and were close to the calibration target value of 8 ms (indicated by horizontal line and asterisk). PRR for HF was similar to control but was increased considerably for models HF + AM compared with HF as shown in Fig. 3*F*. Figure 4 illustrates the time course of the AP, gating variables (*w* and *h*), and three ion currents (*I*_Kr_, *I*_Ks_, and *I*_Ca,L_) from PES cable simulations for the HF + DS model and the arrows show the progressive encroachment of TOP.

### PES and Reentry Induction in 2-D Sheets

Reentry induction simulations were performed in 2-D using the virtual PES protocol for the HF model and all eight HF + AM variants in three sizes of tissue (small, medium, and large), each with a different APD gradient. This gradient was measured from the simulations as the ratio of the longest APD, to the APD at the lower left corner divided by the distance between the sites and is shown in Table 1. By design (see methods) the APD gradients decreased with increasing tissue size and increased in HF + AM models compared with HF.

**Table 1.** Virtual PES simulation results in two dimensions

	Small			Medium			Large		
	Gradient, ms/cm	S2-S4 ERP, ms	EB	Gradient, ms/cm	S2-S4 ERP, ms	EB	Gradient, ms/cm	S2-S4 ERP, ms	EB
**HF**	6.5	**235, 195**	**2**	5.5	**230, 195**	**VT**	4.6	**235, 195, 185**	**VT**
HF + AM1	8.3	300, 285, 280	0	7.5	300, 285, 275	0	6.4	300, 285, 275	0
HF + AM2	9.7	300, 290, 280	0	8.0	305, 285, 280	0	6.0	300, 285, 280	0
HF + AM3	9.7	305, 295, 300	0*	7.7	310, 295, 285	0*	6.4	320, 300, 290	0
HF + AM4	10.0	305, 300, 285	0*	8.0	305, 295, 275	0*	7.3	310, 290, 285	0*
HF + AM5	9.4	305, 290, 280	0	7.6	305, 285, 280	0	5.7	305, 290, 275	0
HF + AM6	9.5	305, 285, 280	0*	7.8	300, 285, 275	0	6.7	300, 290, 280	0
HF + AM7	9.0	305, 290, 280	0*	7.4	305, 290, 280	0	6.4	305, 290, 280	0
HF + AM8	9.6	**305, 285, 285**	**1**	7.7	300, 285, 275	0*	6.9	305, 285, 280	0*

PES, programmed electrical stimulation; ERP, effective refractory period; EB, extra beat; HF, heart failure; VT, ventricular tachycardia; AM1–8, amiodarone models 1–8. *wave break. Boldface indicates reentry induction.

**Figure 5.**
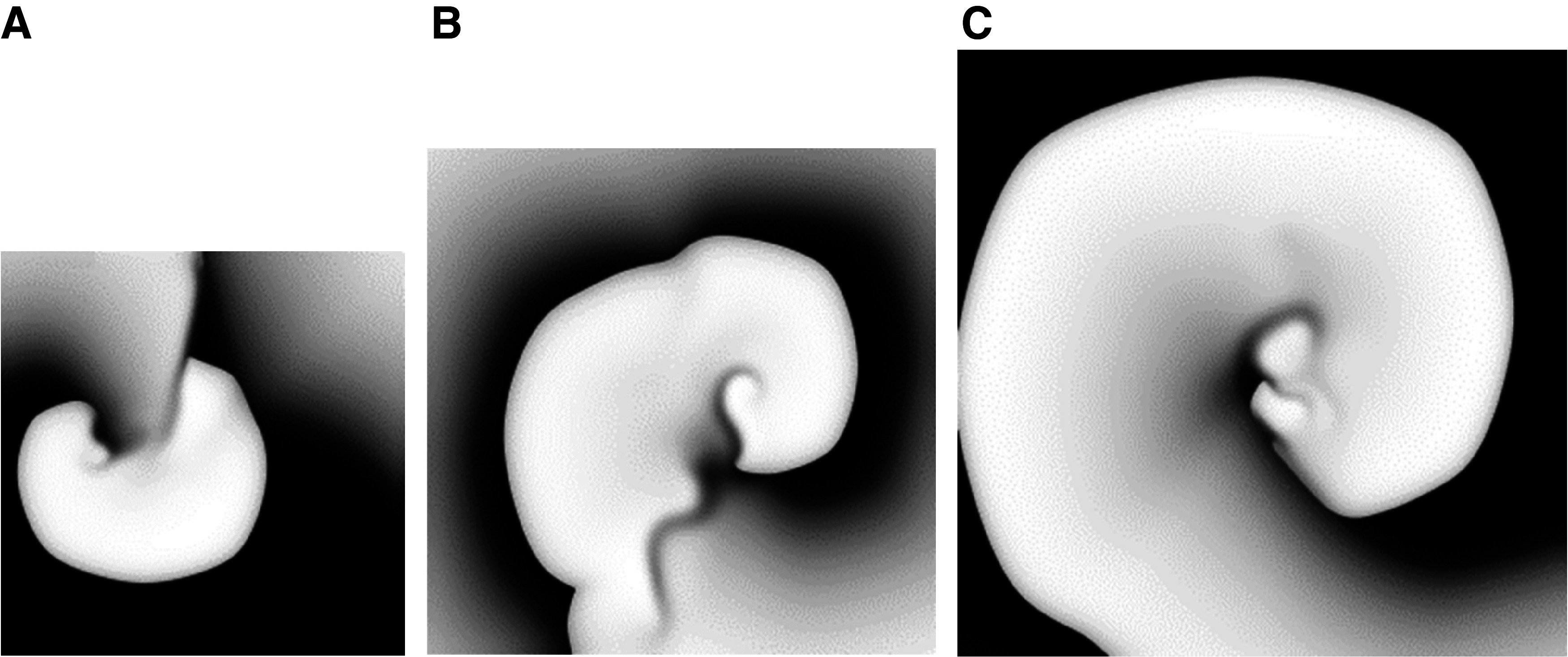
Snapshots from the heart failure (HF) model resulting from programmed electrical stimulation (PES) simulations in the presence of an action potential duration (APD) gradient in various tissue sizes: small (*A*), medium (*B*), and large (*C*).

Reentry was induced in the HF model in all tissue sizes as shown in Table 1 and Fig. 5. Reentry terminated after two rotations in the smallest tissue size but was sustained for the duration of the simulations (2 s) in the medium and large sheets for HF. Reentry occurred after S3 for the small and medium sheets while it occurred after S4 in the large size. Sustained reentry was maintained largely by a single rotating wave throughout the simulations.

**Figure 6.**
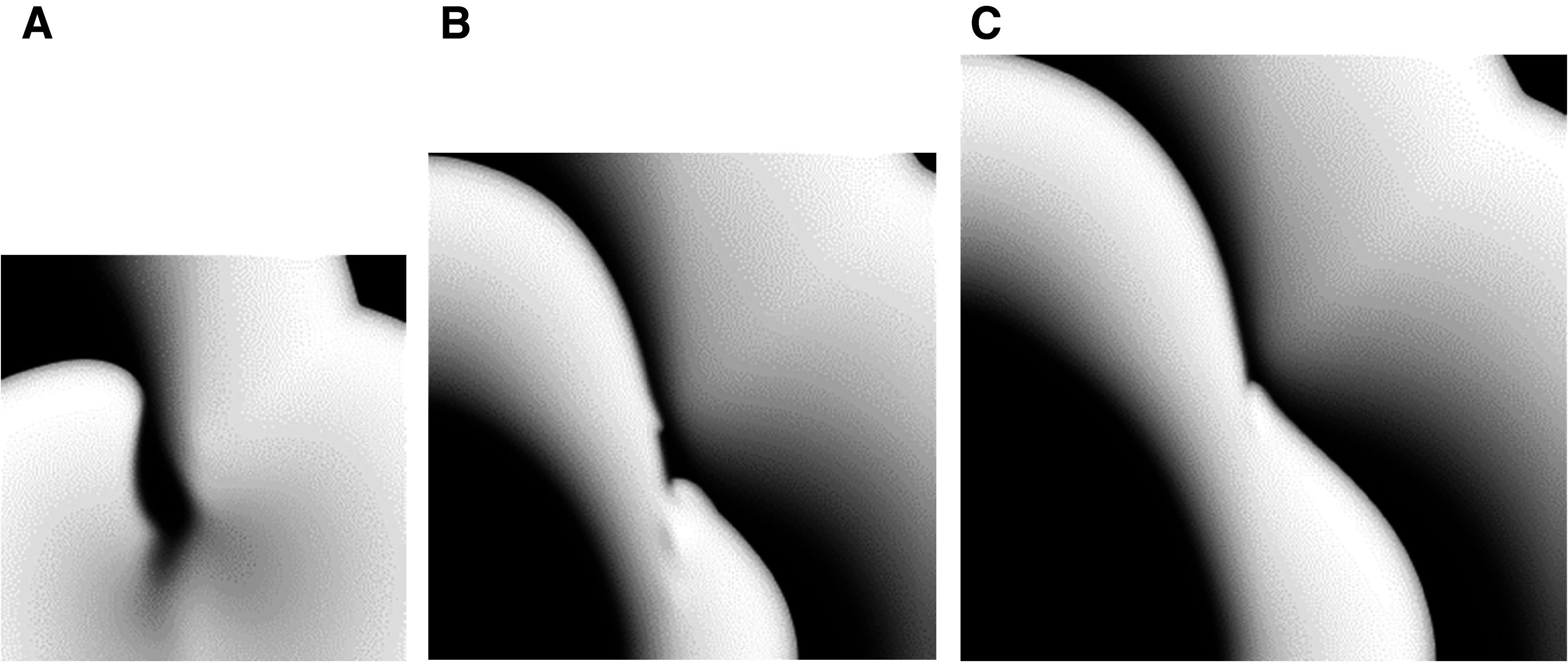
Snapshots from the heart failure (HF) + amiodarone (AM)-8 model resulting from programmed electrical stimulation (PES) simulations in the presence of an action potential duration (APD) gradient in various tissue sizes: small (*A*), medium (*B*), and large (*C*).

Only one of the 24 HF + AM simulations (HF + AM8 in small tissue size) resulted in reentry formation which lasted only for a single rotation. Snapshots of activity for the HF + AM8 model in all tissue sizes are shown in Fig. 6. Wave break without reentry formation occurred in the HF + AM8 model in medium and large tissues as indicated by the asterisk in Table 1; wave breaks (but not reentry) also formed for HF + AM4, HF + AM5, HF + AM7, and HF + AM8 model variants, but never for models HF + AM2, HF + AM3, and HF + AM6.

### Results of Altering TauH in AM and HF + AM Models

**Figure 7.**
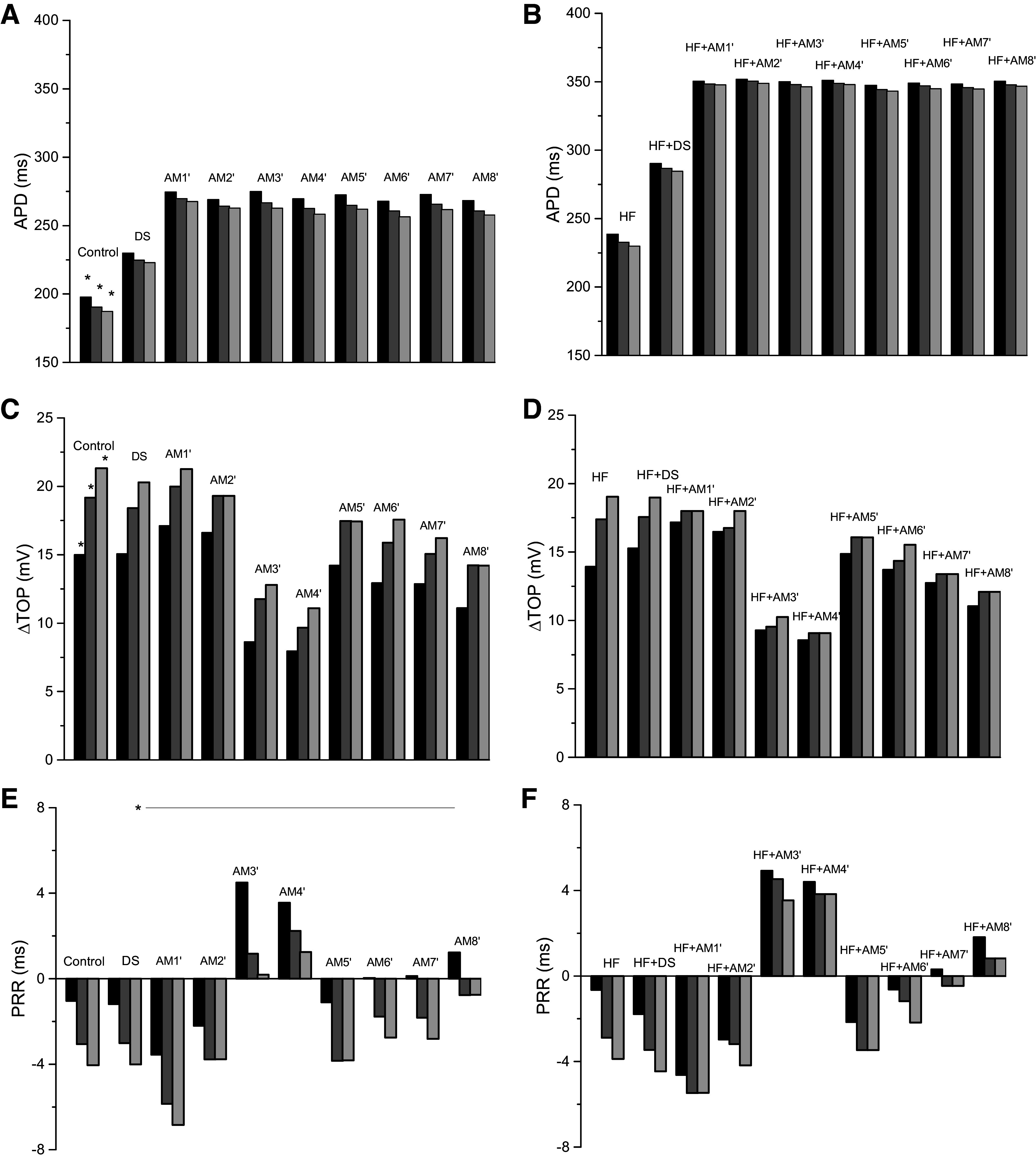
Results for d-sotalol (DS) and for heart failure (HF) and HF + amiodarone (AM) models with altered time constant of *I*_Na_ inactivation (TauH): results from cellular action potential simulations: control, DS, and 8 AM′ models (*left*) and HF, DS, and 8 HF + AM′ models (*right*). *A–F*: action potential duration (APD; *A* and *B*), change in take-off potential (ΔTOP; *C* and *D*), and post-repolarization refractoriness (PRR; *E* and *F*). Corresponding results from cable simulations are presented in Supplemental Figs. S2 and S3.

**Figure 8.**
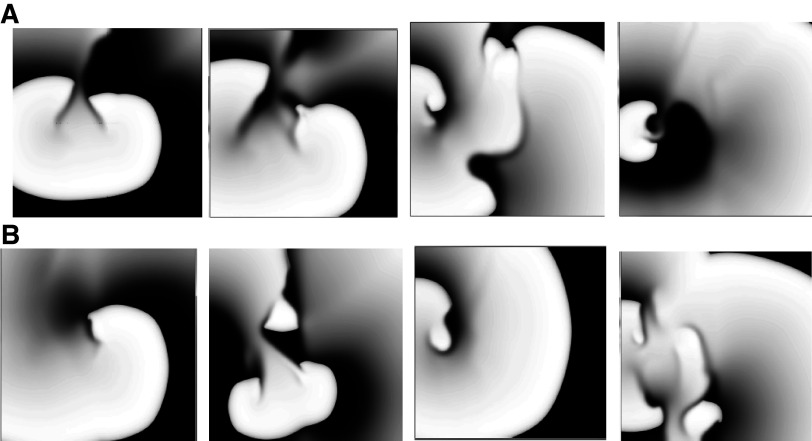
Snapshots from the heart failure (HF) + amiodarone (AM)′ models: HF + AM1′ to HF + AM4′ (*A*) and HF + AM5′ to HF + AM8′ (*B*).

We removed the slowing of the recovery of *I*_Na_ inactivation in the HF + AM models such that parameter TauH values were twice that of the control model (which matches the change for the DS model); these models are referred to as HF + AM′ and were not calibrated to the PRR values of patients. The AM′ (*left*) and HF + AM′ (*right*) cell model results are shown in Fig. 7; the corresponding plots for cable simulations are shown in Supplemental Fig. S2 and the CV values are presented in Supplemental Fig. S3. When compared with AM and HF + AM models (see Fig. 3 and Supplemental Fig. S2), APD results for AM′ and HF + AM′ were similar for both cell and cable simulations. When compared with AM and HF + AM models, ΔTOP, and PRR results were significantly reduced for both cell and cable simulations (although PRR values for AM3′ and AM4′ remained positive). In addition, reduction of TauH to twice control values (compared with AM models) restored the ability of PES to initiate reentry in HF + AM′ models in 2-D tissue (20 cm size) as shown in Table 2; the measured APD gradients for to HF + AM′ models were slightly larger than HF + AM models (see Table 1). Snapshots of activity for the HF + AM′ models are shown in Fig. 8.

**Table 2.** DS and the effect of parameter TauH: virtual PES simulation results in two dimensions

	Medium		
2 × TauH	Gradient, ms/cm	S2-S4 ERP, ms	EB
**HF**	5.5	**230, 195**	**VT**
HF + DS	7.6	**235, 195, 185**	**VFVT**
HF + AM1′	7.6	**295, 270, 260**	**NSVF**
HF + AM2′	8.1	**295, 270, 260**	**VT**
HF + AM3′	8.7	**310, 285, 290**	**NSVF**
HF + AM4′	7.4	**305, 285, 275**	**NSVF**
HF + AM5′	7.3	**300, 275, 265**	**VT**
HF + AM6′	7.9	**295, 270, 260**	**NSVF**
HF + AM7′	7.5	**305, 280, 270**	**NSVF**
HF + AM8′	8.1	**300, 275, 255**	**VF**

DS, d-sotalol; TauH, time constant of *I*_Na_ inactivation; PES, programmed electrical stimulation; ERP, effective refractory period; EB, extra beats; HF, heart failure; VFVT, very fast ventricular tachycardia (VT); NSVF, nonsustained ventricular fibrillation; AM1–8, amiodarone classes 1–8. Boldface indicates reentry induction.

### Results of DS Model

Recall that the DS model was calibrated to the PRR values of patients, which were similar to the control (Fig. 6, *left*). When compared with control and HF models, APD results were similar for both cell and cable simulations. When compared with AM and HF + AM models, APD was significantly increased consistent with its class III properties in both cell and cable simulations (see Fig. 7 and Supplemental Fig. S3). The values of ΔTOP and PRR for DS and HF + DS models were similar to control and HF models, respectively, suggesting that ΔTOP and PRR values are not sensitive to class III effects for both cell and cable simulations. ΔTOP and PRR for DS and HF + DS models were also similar to AM′ and HF + AM′ models, respectively with the exception of AM3′ and AM4′ models. Results from PES simulations demonstrate that, unlike AM, DS did not prevent the induction of reentry in 2-D tissue (20 cm size) as shown in Table 2.

### Role of *I*_Ca,L_

Although *I*_Na_ is the primary current driving AP depolarization, *I*_Ca,L_ may play a role during compromised propagation (47). In the cell simulations, active depolarization was defined as peak *I*_Na_ more negative than −100 µA/cm^2^, therefore only the cable simulations provide meaningful insight into the relative role of *I*_Ca,L_ and *I*_Na_ during active depolarization. Since the *I*_Ca,L_ current is active from ∼ −30 mV to ∼ –5 mV in our model, and this is significantly more positive than the most elevated TOP in our study, it is unlikely to contribute to the genesis of active propagation. To confirm this hypothesis, we measured the peak *I*_Na_ and *I*_Ca,L_ currents during S1 and S4 beats in all cable simulations. Peak *I*_Na_ was significantly decreased by 32% for control; 36% for HF, 22% for HF + AM3; 18% for HF + DS while peak *I*_Ca,L_ was much less affected: 78% for control; 63% for HF, 82% for HF + AM3; 72% for HF + DS. These results confirm that the decrease in peak *I*_Na_ (and not *I*_Ca,L_) is responsible for the alterations in TOP and PRR in this study although the role of *I*_Ca,L_ during propagation along the cable (which could be highly dynamic) is a possibility.

## DISCUSSION

In this study, we elucidate the probable mechanism of the antiarrhythmic action of AM in patients with HF. Our results suggest that AM prevents the formation of reentrant waves at fast rates primarily via its effects on the recovery of excitability as evidenced by the increase of PRR (see Fig. 3*D*) and the elimination of the elevation of TOP (see Figs. 3*D* and 4). Although both class III effects and class I effects of AM were implemented in the model, the following results indicate the prevention of reentry initiation resulted from its class I properties (not class III): decreasing the *I*_Na_ inactivation time constant (TauH) in the AM model to correspond to the values of the DS (i.e., model AM′) restored the ability to induce reentry (see Table 2 and Figs. 7 and 8); the values of ΔTOP and PRR for DS and the results for HF + DS models were similar to HF, suggesting that ΔTOP and PRR values are not sensitive to class III effects.

We believe that AM’s ability to produce PRR, with concomitant removal of the elevation of TOP, acts to decrease front-tail interactions and prevent reentry initiation. This idea is consistent with the clinical findings of Osaka et al. (9) who demonstrated that AM suppressed VT/VF concomitant with an increase in the shortest diastolic interval to produce a ventricular response. In addition, IV AM has been shown effective in terminating VT in the acute clinical setting (48) despite eliciting only ∼8% APD prolongation compared with 29% for chronic AM (18) suggesting that AM’s class I effects are responsible for acute VT termination. Our results are also consistent with experimental findings of AM in rabbit (7) and dog (49) as well as with antiarrhythmic outcomes corresponding to PRR induced by other drugs in rabbit ventricles (50, 51) and dog atria (52).

Our results provide mechanistic insight into AM-induced PRR; specifically, our sensitivity analysis (see Supplement data) indicates that AM’s effects on parameters Eh and TauH have a large effect on PRR and that AM’s effect on g_Na_ and gK1 affect TOP. We believe that AM alters the voltage and time dependence of *I*_Na_ inactivation in such a way as to fundamentally alter the normal recovery of excitability and produce PRR. More precisely, the major effect of AM is to delay the recovery of inactivation of *I*_Na_ (via an increase in TauH) such that excitability is restored after complete repolarization of the AP thus increasing ERP and producing PRR. The voltage dependence of TauH plays a crucial role in the recovery of excitability as described previously (12, 53), and as such its modification by AM is important, and is shown in Supplemental Fig. S4. Briefly, TauH is very small (< 1 ms) during the plateau for all models, which is necessary to ensure rapid closure of *I*_Na_ immediately after AP depolarization, however, the values of TauH are increased substantially during phase 3 and AM delays the restoration of excitably by slowing the kinetics of *I*_Na_ inactivation (i.e., increasing the time constant of the h-gate, TauH from control values of ∼7 ms to between 50 and 110 ms). Studies using the voltage-clamp and sucrose-gap techniques (to quantify the highly nonlinear voltage and time dependence of ion currents) have established that the recovery of excitability is a fundamental property of cardiac tissue resulting from the voltage and time dependence of *I*_Na_ near membrane potential values of −70 mV and *I*_Ca,L_ near –50 mV (54–57). Mason et al. (58) suggest that the mechanism of AM-induced PRR is due to a preferential blockade of cardiac sodium channels in the inactivated state, which explains how AM’s alteration of *I*_Na_ inactivation produces PRR. We believe that AM-induced PRR acts to alter the “graded” nature of *I*_Na_ recovery that results in slow propagation near the ERP in normal tissue and steepened activation time restitution, which is known to be protective against conduction block and reentry (59–61).

The initiation and maintenance of reentry involves not only dynamic front-tail interactions but also more global factors, such as tissue size (25), APD dispersion (62, 63), and critical curvature (64). These factors have yet to be robustly measured and quantified in patients with HF. Our approach was to study the robustness of our simulation results of reentry induction via the use of three tissue sizes (length: 16, 20, and 24 cm) each with their own nearly linear APD gradient, ranging from 4.6 ms/cm in the large tissue size to 6.5 ms/cm in the small size. Linear APD gradients over large distances are probably unlikely in patients with HF (23, 24), however, they represent a simplified pattern, which is easier to study compared with the alternative of a wide range of sizes and numbers of individual heterogeneities. The APD dispersion in the HF + AM models was increased compared with HF alone (see Table 2), which we believe represents a conservative condition since chronic AM has been shown to decrease APD dispersion (29, 65) or have no effect (66, 67), although acute AM has been shown to increase APD dispersion (67).

There are several limitations in this study. First, we did not address the potential effect of AM on early afterdepolarization (EAD) development and Torsade de Pointes (TdP) arrhythmias. Despite its categorization as a class III antiarrhythmic drug, AM has been shown in many studies to be extremely unlikely to cause TdP and is considered safe by many guidelines (68–71). Furthermore, drug-induced TdP is facilitated by bradycardia or pauses, and suppressed by tachycardia or PES. In addition, the late sodium current is extremely small compared with the rapid sodium current and therefore is not relevant to our PES study, but would be important in the study of EADs in HF. Second, our model does not include several important cellular processes such as transmembrane pumps and exchangers, intracellular calcium handling, and changes in intracellular ion concentration. In addition, most time constants are not voltage-dependent, and some gates were assumed to be instantaneous, therefore some of the fine details of the voltage-clamp experimental data will not be reproduced by this model. Third, because of the complex metabolism and pharmacokinetics of AM (22, 67, 72), the specific time course and active metabolites corresponding to the AM alterations, we propose here remain unknown. AM is characterized by complex pharmacokinetics, which makes pinpointing the exact contributions to either class I or class III effects difficult. An IV load is used, often successfully, to terminate VT even if an IV push with lidocaine was unsuccessful. There is often no or minimal QT prolongation present after acute IV AM (18, 44, 73). On the other hand, chronic therapy requires a prolonged loading phase because of the large lipophilic reservoir (or volume of distribution) that needs to be filled before substantive cardiac tissue concentrations can be achieved. During this time, AM is metabolized in the liver into *N*-desethylamiodarone (DEA) which, according to consensus, is the actual APD prolonging (class III) agent (22, 72, 74). Thus, the sodium channel-modulating effect may be attributable largely to AM and the APD prolonging effect predominantly to DEA. However, it is unknown to what extent DEA has sodium channel effects. Fourth, our model incorporates a rapid inactivation gate (*h*) but not a separate slow inactivation gate; instead, the values of inactivation kinetics are voltage-dependent and the recovery of inactivation during repolarization is a very complex function of all inactivation parameters as well as the diastolic value of intracellular calcium, and the control model was calibrated to the PRR values during PES as described in Ref.^12^. Fifth, the HF + AM models were constructed assuming that the effects of HF and AM effects on individual model parameters are independent and multiplicative or additive, which may not be the case. The effects of both HF and AM on ionic currents in human cardiac myocytes are known to be complex and multifactorial, therefore detailed characterization of such effects, as well as their uncertainty, is required for a comprehensive understanding of our results; however, most voltage-clamp data are limited to controlled nonprimate animal experiments. Our combinatorial approach to AM modeling is meant to be an alternative approach. In fact, our preliminary simulations included 5 HF model variants in addition to AM combinatorial models, but it appeared that the results of HF models were very similar. Incidentally, the modulated and guarded receptor hypotheses (75) have been implemented similarly to what was done here, in which individual model parameters are derived from experimental data; however, the specific details of the translation of experimental results to model parameters differ.

Here, we present a likely mechanism of the antiarrhythmic effect of AM in patients with HF. Our computational modeling approach allowed us to integrate the most pertinent clinical and benchtop data, and our modeling framework provided a robust way to investigate the variation and dissimilar voltage-clamp results. Our results are robust to a range of tissue sizes and APD gradients and implicate the time constant of the recovery of *I*_Na_ inactivation’s effect on PRR as a necessary component of AM’s ability to prevent reentry. The results from this study can be used to inform patient-specific computational modeling (76, 77) that once validated, can predict the effects of AM in patients with HF, for example, using measured clinical APD gradients (24). In addition, the insights provided into the relationship among *I*_Na_ recovery, PRR, and reentry induction by means of our computational modeling approach have the potential to guide the future development of antiarrhythmic drugs that replicate AM’s efficacy without untoward side effects.

## DATA AVAILABILITY

Data will be made available upon reasonable request.

## SUPPLEMENTAL DATA

10.6084/m9.figshare.24065610 Supplemental Tables S1–S3 and Supplemental Figs. S1–S4: https://doi.org/10.6084/m9.figshare.24065610.

## DISCLAIMERS

The mention of commercial products, their sources, or their use in connection with material reported herein is not to be construed as either an actual or implied endorsement of such products by the Department of Health and Human Services.

## DISCLOSURES

No conflicts of interest, financial or otherwise, are declared by the authors.

## AUTHOR CONTRIBUTIONS

R.A.G. and M.R.F. conceived and designed research; R.A.G. performed experiments; R.A.G. analyzed data; R.A.G. and M.R.F. interpreted results of experiments; R.A.G. prepared figures; R.A.G. drafted manuscript; R.A.G. and M.R.F. edited and revised manuscript; R.A.G. and M.R.F. approved final version of manuscript.
